# New *Crepidotus* (Agaricales, Crepidotaceae) findings in China: *Crepidotuscinereofuscus* sp. nov. and record of *C.stenocystis*

**DOI:** 10.3897/BDJ.13.e153245

**Published:** 2025-07-14

**Authors:** Xiaopo Yang, Menghui Han, Tiezheng Wei, TieZhi Liu

**Affiliations:** 1 College of Chemistry and Life Sciences, Chifeng University, Chifeng 024000, China College of Chemistry and Life Sciences, Chifeng University Chifeng 024000 China; 2 School of Horticulture, Ludong University, Yantai 264025, China School of Horticulture, Ludong University Yantai 264025 China; 3 State Key Laboratory of Mycology, Institute of Microbiology, Chinese Academy of Sciences, Beijing 100101, China State Key Laboratory of Mycology, Institute of Microbiology, Chinese Academy of Sciences Beijing 100101 China

**Keywords:** Inocybaceae, biodiversity, new taxon, morphology, taxonomy

## Abstract

**Background:**

*Crepidotus* is an essential group of saprotrophic fungi within ecosystems and exhibits a widespread global distribution. In China, approximately 80 species have been discovered, most of them being collected from humid monsoonal climate zones of China.

**New information:**

In this paper, we report on several specimens of *Crepidotus* collected from the Inner Mongolia Autonomous Region, an arid area of China characterised by a continental climate. Morphological and phylogenetic analyses revealed two taxa: the first record for China of *Crepidotusstenocystis* Pouzar and a new species *Crepidotuscinereofuscus* sp. nov. *C.stenocystis* is a wood-inhabiting saprotrophic fungus, belonging to C.subg.Dochmiopussect.Sphaeruli Hesler & Smith because of the presence of clamp connections and subglobose verrucose basidiospores. Phylogenetically, the Chinese sequence of *C.stenocystis* formed a strongly-supported clade with other reliable sequences. *C.cinereofuscus* is a soil-saprotrophic fungus, classified in C.subg.Dochmiopussect.Autochthoni Consiglio & Setti because of its smooth basidiospores and clamp connections. Phylogenetic analyses confirmed that *C.cinereofuscus* is closely related to *Crepidotusclavocystidiatus* M.H. Han, Q. Na & Y.P. Ge, but it is clearly separated and formed a distinct clade. *C.cinereofuscus* is also different from *C.clavocystidiatus* in morphological characteristics. Detailed species descriptions, photographs and line drawings of these taxa are provided. This study enhances our understanding of *Crepidotus* diversity in the Inner Mongolia Autonomous Region, raising the total number of *Crepidotus* species reported from this region to seven.

## Introduction

*Crepidotus* (Fr.) Staude, the type genus of Crepidotaceae, comprises pleurotoid agarics, is characterised by dark-coloured basidiospores ranging from pale yellow to dark brown and contains approximately 400 species worldwide (Hesler and Smith 1965, Senn-Irlet 1995, Aime 2001, Matheny et al. 2006 ,Consiglio and Setti 2008, https://www.indexfungorum.org/Names/Names.asp, accessed on 17 April 2025). The most recent classification of *Crepidotus* was proposed by Consiglio and Setti (2008), who divided *Crepidotus* into two subgenera, C.subg.Crepidotus Hesler & A.H. Sm. and C.subg.Dochmiopus (Pat.) Pilát. They considered C.subg.Crepidotus with clamp connections absent and C.subg.Dochmiopus with clamp connections present (Consiglio and Setti 2008). Additionally, C.subg.Sphaeruli Hesler & A.H. Sm. established by Hesler and Smith in 1965, was combined into C.subg.Dochmiopus (Hesler and Smith 1965, Consiglio and Setti 2008). Generally, species of C.subg.Crepidotus possess smooth basidiospores and no clamp connections, while those of C.subg.Dochmiopus exhibit ornamented basidiospores and clamp connections. However, several exceptional species exhibiting both clamp connections and smooth basidiospores have been identified. Consiglio and Setti (2008) classified these species within C.subg.Dochmiopussect.Autochthoni Consiglio & Setti. Currently, taxonomic research within C.subg.Dochmiopussect.Autochthoni remains limited due to the small number of recognised species. Additional specimens and potential new taxa are required to elucidate the phylogenetic relationships.

*Crepidotus* is widely distributed across diverse ecosystems, including forests, grasslands and even urban environments. Basidiomata ranging from white to pale yellow are commonly encountered, particularly amongst members of the *C.variabilis*-group and the *C.mollis*-group. In contrast, basidiomata exhibiting grey to greyish-brown colours are rare. Within China, specimens characterised by non-globose basidiospores are more frequently observed, whereas those with globose basidiospores are comparatively uncommon. To date, approximately 80 species of *Crepidotus* have been recorded in China (Liu 1995, Wei and Yao 2009, Ge 2017, Zhang P 2017, Ge et al. 2017, Ge and Bau 2020, Tao 2021, Zhang et al. 2022, Na et al. 2022, Liu et al. 2024, Han et al. 2024). Most previous studies have focused on regions influenced by humid monsoonal climates, leaving specimens from arid continental climatic zones relatively understudied. The Inner Mongolia Autonomous Region, with a continental climate, is characterised by aridity and extensive desert landscapes. In recent years, we have collected over 100 *Crepidotus* specimens from this region. A portion of these specimens has been examined in detail and five known species have been identified, based on morphological and molecular evidence: *C.cesatii* (Rabenh.) Sacc., *C.crocophyllus* (Berk.) Sacc., *C.mollis* (Schaeff.) Staude, *C.subverrucisporus* Pilát and C. *dentatus* T. Bau & Y.P. Ge (Shi 2021, Bai et al. 2022). Further studies are underway on the remaining collections, which may reveal additional diversity within this genus. In the present study, we document *C.stenocystis* as a first record for China and describe a new species *C.cinereofuscus* sp. nov. These findings contribute to our understanding of *Crepidotus* diversity within the Inner Mongolia Autonomous Region, increasing the total number of known species in the region to seven.

## Materials and methods

### Specimens and Morphology

The four specimens examined in this study were collected from the Inner Mongolia Autonomous Region and Shandong Province in 2007, 2022 and 2023. Dried specimens have been deposited in the Mycological Herbarium of Chifeng University (CFSZ) and the Fujian Academy of Agricultural Sciences (FFAAS). Freshly collected specimens were dried with portable dryers at 40°C (Stöckli, Netstal) and then sealed and stored with allochroic silica gel. Macroscopic characteristics were described by field records based on fresh basidiomata. Colour descriptions follow the standards and nomenclature established by Ridgway (Ridgway 1912). Microscopic character observations were performed on dried material rehydrated in 5% potassium hydroxide (KOH) solution examined using a Lab A1 microscope (Carl Zeiss AG, Jena, Germany). For each specimen, at least 20 measurements were recorded for each structure, including basidiospore, basidia, cheilocystidia and pileipellis hyphae. The notation [a/b/c] used at the beginning of each basidiospore description, indicates that the a basidiospores from b basidiomata of c specimens were measured. Measured dimensions (length × width) are presented as (d)e-f-g(h) × (i)j-k-l(m), in which d is the smallest length, e-g represents the range of at least 90% of values, f is the average length and h is the largest length; same expression is used for width (i-m). The ratio of basidiospores length to width is represented by Q, and Q_m_ denotes the mean of these ratios ± the sample standard deviation (Na et al. 2024). Basidiomata and microscopic structures were illustrated manually and the line drawings on the tracing paper were digitised into TIFF format using a Canon LiDE120 scanner (Canon, Tokyo, Japan). Subsequently, the scanned images were post-processed in Adobe Photoshop 2020 for clarity enhancement, including background cleaning, cropping and minor adjustments to brightness and contrast. Final image layout was also completed using the same software.

### DNA extraction, amplification, sequencing and Phylogenetic analysis

In this study, only the ITS region was utilised for phylogenetic analysis, as the LSU region and other protein-coding region sequences for *Crepidotus* remain scarce and, therefore, provide limited utility for phylogenetic analyses. Moreover, the ITS region has been demonstrated to be sufficient for species-level identification within *Crepidotus* (Na et al. 2022, Liu et al. 2024). Amplification of the ITS region was performed using the primer pair ITS1 and ITS4 (White et al. 1990). PCR amplification was conducted using the following thermal cycling conditions: 94°C for 5 min, followed by 30 cycles of 94°C for 30 s, 50°C for 30 s and 72°C for 30 s, with a final extension at 72°C for 10 min. All PCR products were sequenced by the Beijing Genomics Institute. Sequencing results were checked with Chromas 2.6.5 (https://chocolatey.org/packages/chromas/2.6.5). A total of five sequences were generated from the collected specimens and obtained accession numbers OQ946646 - OQ946648, OQ944135 and PQ432544. The sequence matrix comprised these newly-generated sequences and representative *Crepidotus* sequences downloaded from GenBank (https://www.ncbi.nlm.nih.gov/genbank/, accessed on 23 April 2025). Detailed sequences informations are provided in Table 1. Sequences were aligned using MAFFT v.7 (https://mafft.cbrc.jp/alignment/server/index.html), manually inspected and adjusted in BioEdit v.7.0.4.1 and regions with ambiguous alignment or excessive gaps were trimmed using MEGA 10 (Hall 1999, Katoh et al. 2002, Alzohairy 2011, Katoh and Standley 2016). Phylogenetic analyses were conducted using Bayesian Inference (BI) and Maximum Likelihood (ML). The BI analyses were performed by MrBayes v.3.2.6 and the best-fit substitution model inferred by MrModelTest 2.3 (Ronquist and Huelsenbeck 2003, Nylander et al. 2008). Eight MCMC chains were run for 2,000,000 generations and sampled every 1000 generations. At the end of the run, the average standard deviation of split frequencies was less than 0.01. Tracer v.1.7.2 was used for the visualisation and examination of MCMC trace files from BI analyses (Rambaut et al. 2018). In total, 25% of the samples were discarded as burn-in (Ronquist and Huelsenbeck 2003). The ML analysis was conducted using raxmlGUI v.2.0.1.5 (Mac version; Edler et al. (2021)), which implements RAxML-NG (Kozlov AM et al. 2019), with 1000 bootstrap replicates. The resulting phylogenetic trees were visualised and edited with FigTree 1.4.4 and Adobe Photoshop 2020.

## Taxon treatments

### Crepidotus cinereofuscus

T. Z. Liu, X. P. Yang & T. Z. Wei sp. nov.

B236FF7F-DDD9-57AD-A820-FFFE5459CA47

856213

#### Materials

- **Type status:** Holotype. **Occurrence:** occurrenceID: B32B0132-CF15-59A3-A87C-47869FB3D0F6; **Taxon:** kingdom: Fungi; phylum: Basidiomycota; class: Agaricomycetes; order: Agaricales; family: Crepidotaceae; genus: Crepidotus; subgenus: Dochmiopus; taxonRank: species; **Location:** country: China; stateProvince: Inner Mongolia Autonomous Region; county: Chifeng City; verbatimLocality: Chifeng University; verbatimElevation: 585 m; verbatimLatitude: 42°14′33″ N; verbatimLongitude: 118°54′40″ E; **Identification:** identifiedBy: TieZhi Liu; **Event:** year: 2022; month: July; day: 7; **Record Level:** institutionID: CFSZ; collectionID: CFSZ 24821
- **Type status:** Other material. **Occurrence:** occurrenceID: C95E606E-E92A-5F10-9847-394B136EB38C; **Taxon:** kingdom: Fungi; phylum: Basidiomycota; class: Agaricomycetes; order: Agaricales; family: Crepidotaceae; genus: Crepidotus; subgenus: Dochmiopus; taxonRank: species; **Location:** country: China; stateProvince: Inner Mongolia Autonomous Region; county: Chifeng City; verbatimLocality: Chifeng University; verbatimElevation: 583 m; verbatimLatitude: 42°14′33″ N; verbatimLongitude: 118°54′40″ E; **Identification:** identifiedBy: TieZhi Liu; **Event:** year: 2022; month: July; day: 7; **Record Level:** institutionID: CFSZ; collectionID: CFSZ 24838
- **Type status:** Other material. **Occurrence:** occurrenceID: 55CCEAEB-ECD8-599B-975F-6C14047D92AF; **Taxon:** kingdom: Fungi; phylum: Basidiomycota; class: Agaricomycetes; order: Agaricales; family: Crepidotaceae; genus: Crepidotus; subgenus: Dochmiopus; taxonRank: species; **Location:** country: China; stateProvince: Shandong Province; county: Tai’an City; verbatimLocality: Taishan Natural Sight; verbatimElevation: 1473 m; **Identification:** identifiedBy: Menghui Han, Renxiu Wei, and Qin Na; **Event:** year: 2023; month: August; day: 17; **Record Level:** institutionID: FFAAS; collectionID: FFAAS0367

#### Description

**Figure 1.**
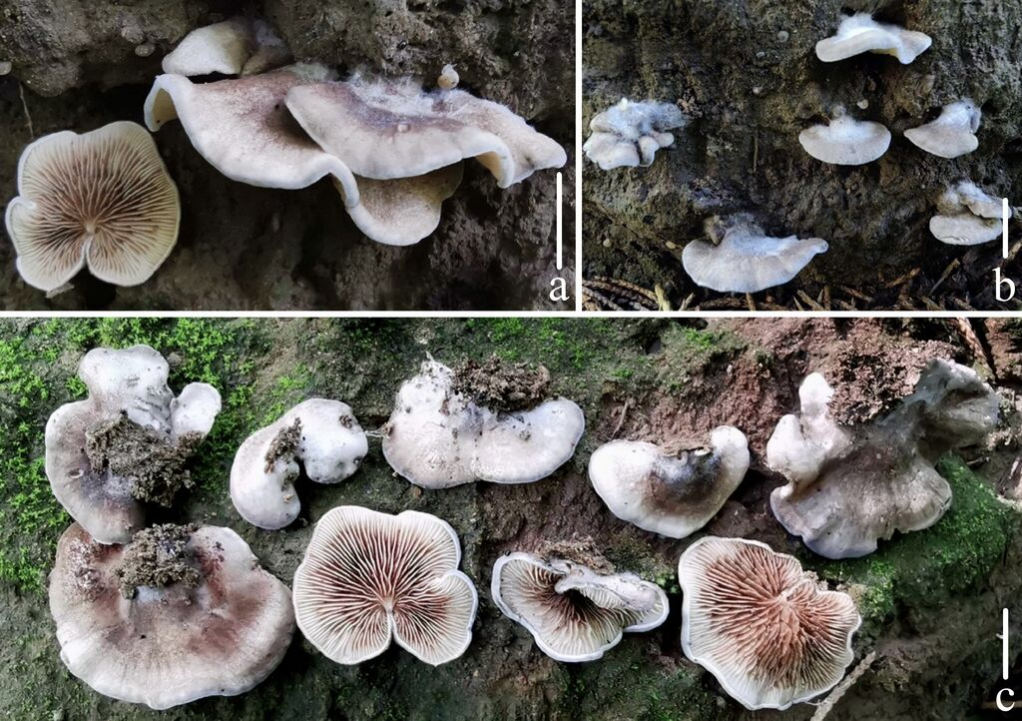
Fresh basidiomata of *Crepidotuscinereofuscus* sp. nov. Bars: **a**–**c** = 10 mm. Photos by Tiezhi Liu.

**Pileus** 10–35 mm, attached laterally, sometimes dorsally to the substratum, ungulate to conchoid when young, *Lavender Grey (XLIII49'''*f*) to Greyish Lavender (XLIII57'''*f*), when mature flabelliform, semicircular to petaloid, plane, at the centre Dark Slate-Violet (1) (XLIII7'''*k*), Deep Purplish Grey (LIII67'''''*i*) to Light Neutral Grey (LIII NEUTRAL GREY*b*), gradually paler from centre to margin, margin White (LIII), margin incurved and glabrescent in all stages; pubescence white, dense near the point of attachment, gradually sparse towards the edge when young, when mature, few and scattered, more or less forming scales; dry, not hygrophanous, non-striated; **Lamellae** 15–25 mm, *L* = 9–16, *l* = 1–7, adnexed to adnate, subventricose, edge smooth; White (LIII) when young, becoming Ivory Yellow (XXX21''*f*) to Brownish-Olive (XXX19''*m*), darker in the centre and paler to the margin when mature. **Stipe** present, cylindrical, at times as a lateral knob or covered by lamellae. **Context** thin (< 5 mm thick), but thicker near the stipe, White (LIII). **Odour** and **taste** not distinctive (Fig. 1).

**Figure 2.**
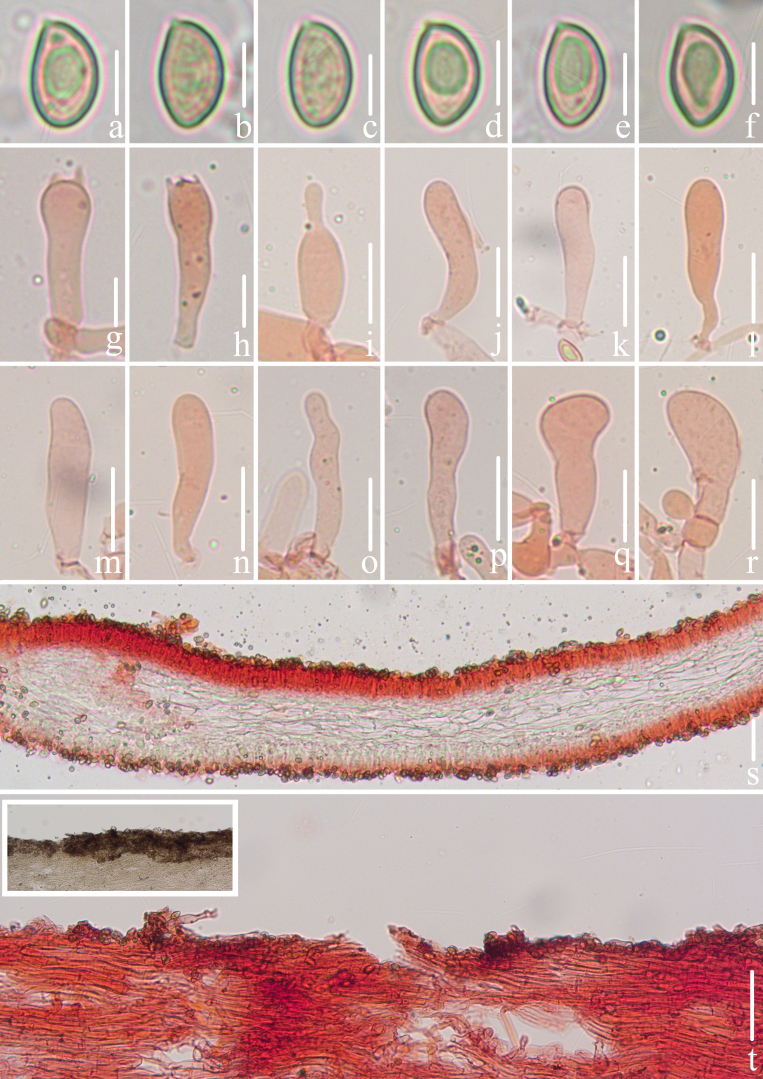
Microscopic features of *Crepidotuscinereofuscus* (CFSZ 24821, Holotype). **a**–**f** Lateral view of basidiospores; **g**, **h** Basidia; **i**–**r** Cheilocystidia; **s** Lamellae trama; **t** Pileipellis. Bars: **a**–**f** = 5 μm; **g**–**h** = 10 μm; **i**–**r** = 20 μm; **s**–**t** = 50 μm. Structures on **a**–**f** were rehydrated in 5% KOH aqueous solution and **g**–**t** were stained in 1% Congo red aqueous solution. Anatomical examination by Menghui Han.

**Figure 3.**
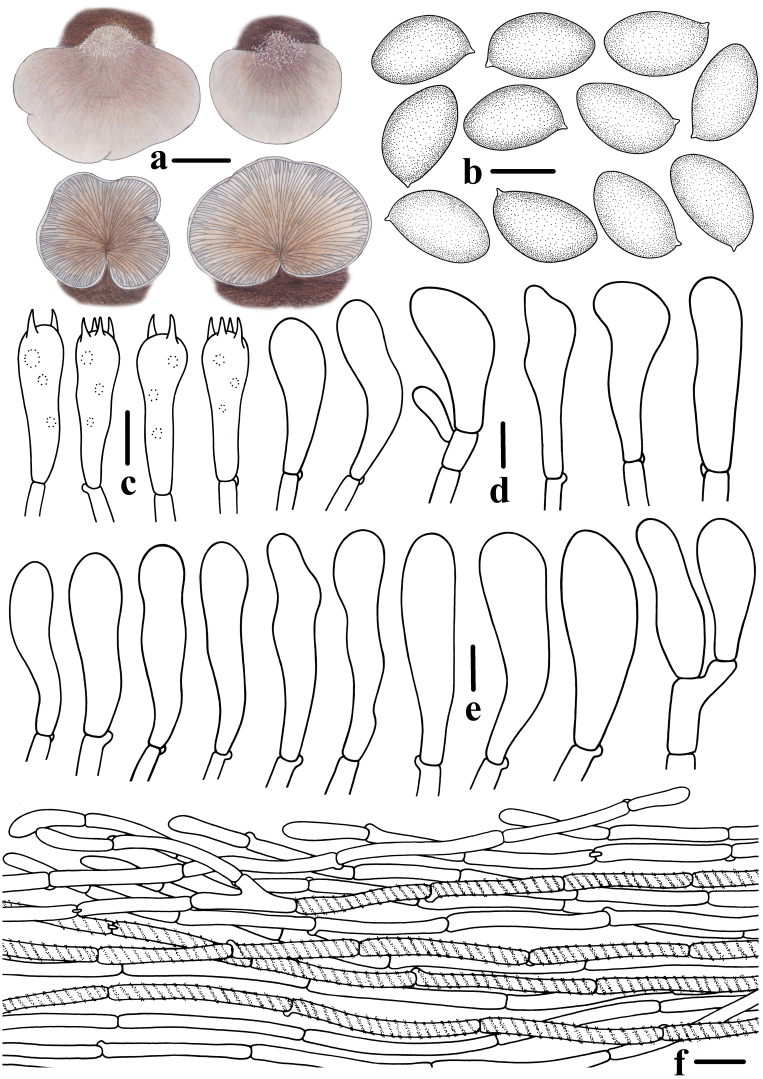
Morphological features of *Crepidotuscinereofuscus* (CFSZ 24821, Holotype). **a** Basidiomata; **b** Basidiospores; **c** Basidia; **d**, **e** Cheilocystidia; **f** Pileipellis. Bars: **a** = 10 mm; **b** = 5 μm; **c**–**e** = 10 μm; **f** = 20 μm. Drawing by Menghui Han.

**Basidiospores** [167/3/2] (6.9)7.4–8.0–8.7(9.3) × (4.3)4.5–4.8–5.4(5.8) μm, Q = (1.52)1.55–1.76(1.86), Q_m_ = 1.66 ± 0.065, [HOLOTYPE [128/2/1] (7.1)7.6–8.0–8.6(9.3) × (4.3)4.5–4.8–5.4(5.8) μm, Q = (1.52)1.56–1.76(1.78), Q_m_ = 1.66 ± 0.064], ellipsoid to oblong in lateral view, ellipsoid to ovoid in frontal view, yellow to ochre in 5% KOH aqueous solution, smooth, slightly thick-walled, sometimes with granular contents (under oil). **Basidia** 24–32 × 7.2–8.7 μm, clavate, 4–spored, rarely 2–spored, sterigmata 2.2–5.2 μm long, thin-walled (< 0.5 μm thick), hyaline. **Pleurocystidia** absent. **Cheilocystidia** 31–50 × 6.8–15.7 μm, clavate, apex rounded, occasionally segmented and bifurcate, middle portion more or less ventricose, constricted to base, hyaline, thin-walled (< 0.5 μm thick). **Pileipellis** a rectocutis, composed of densely arranged cylindrical hyphae, parallel, 4.1–6.7 μm wide, thin- to thick-walled (0.3–0.9 μm), grey extracellular pigment deposits are present in the upper layer, a few hyphae encrusted, non-gelatinised. **Lamellae trama** subregular, composed of subparallel cylindrical hyphae, 7–15 μm diam., subregular, non-gelatinised. Clamp connections present in all tissue (Figs 2, 3).

#### Diagnosis

Pileus dusky grey to purplish-grey, covered with white pubescence, more or less forming scales, not hygrophanous, non-striated; basidiospores ellipsoid to oblong, smooth, clamp connections present. Differs from *C.occidentalis* Hesler & A.H. Sm. by dusky grey to purplish-grey pileus, lamellae edge smooth and wider cheilocystidia.

#### Etymology

The epithet “*cinereofuscus*” combines the Latin adjective “*cinereus*” and “*fuscus*”. This name describes the colour of the pileus, which is dusky grey to purplish-grey.

#### Ecology

Solitary or scattered on soil or soil-covered rock surface.

#### Notes

According to the classification proposed by Consiglio and Setti, *C.cinereofuscus* is classified into C.subg.Dochmiopussect.Autochthoni, based on its smooth basidiospores and the presence of clamp connections (Consiglio and Setti 2008). This section currently comprises seven species: *C.autochthonus* J.E. Lange, *C.wasseri* Kapitonov, Biketova, Zmitr. & Á. Kovács, *C.novae-zealandiae* Pilát, *C.tortus* A.M. Kumar & C.K. Pradeep, *C.trichocraspedotus* T. Bau & Y.P. Ge, *C.occidentalis* and *C.lamellomaculatus* M.H. Han, Q. Na, Y.P. Hu & Y.P. Ge. Amongst these, only *C.occidentalis* and *C.autochthonus* exhibit a slightly grey pileus, while the remaining species are characterised by white to pale yellow pileus, clearly distinguishing them from *C.cinereofuscus*, which has a silver-grey to pale brownish-grey pileus (Hesler and Smith 1965, Senn-Irlet 1995, Consiglio and Setti 2008). Additionally, *C.occidentalis* differs from *C.cinereofuscus* by its fimbriated lamellae edge, wider cheilocystidia and a cutis pileipellis composed of long, straight and slender hyphae (Hesler and Smith 1965, Krisai-Greilhuber et al. 2002). *C.autochthonus* can be distinguished by its gelatinised pileipellis and cream-white, light yellow to silvery-grey pileus, which is lighter in colour than that of *C.cinereofuscus* (Consiglio and Setti 2008, Ge 2017). Amongst the remaining five species, *C.cinereofuscus* can be readily differentiated not only by its distinct pileus colouration, but also by its microscopic characteristics. Microscopically, *C.wasseri* and *C.lamellomaculatus* possess narrowly lageniform cheilocystidia with ventricose middle part, whereas *C.tortus* and *C.trichocraspedotus* exibit irregularly strangulated, vine-shaped cheilocystidia (Ge and Bau 2020, Kapitonov VI 2021, Kumar et al. 2022, Han et al. 2024). The pileipellis of *C.wasseri* and *C.trichocraspedotus* are a trichoderm, *C.lamellomaculatus* is a tomentum, *C.tortus* is a cutis, while *C.cinereofuscus* is a rectocutis pileipellis (Ge and Bau 2020, Kapitonov VI 2021, Kumar et al. 2022, Han et al. 2024). In addition, *C.novae-zealandiae* has a gelatinised pileus and a fimbriated lamellae edge, whereas *C.cinereofuscus* lacks gelatinisation and possesses a smooth lamellae edge (Horak 2018). Phylogenetically, *C.cinereofuscus* is closely related to *C.iqbalii* A. Izhar, Usman & Khalid and *C.subfulviceps* (Murrill) Aime, Vila & P.-A. Moreau. However, both *C.iqbalii* and *C.subfulviceps* can be clearly distinguished by their distinct stipe and yellow coloured pileus, contrasting with the sessile, silver-grey to pale brownish-grey pileus of *C.cinereofuscus*.

The specimen *FFAAS*0367, collected from Shandong Province, differs from those collected in the Inner Mongolia Autonomous Region by its basidiospores dimensions. The basidiospores of *FFAAS*0367 are (6.7)6.9–7.7–8.5(8.8) × (4.7)4.8–5.3–6.1(6.2) μm, Q = (1.33)1.36–1.55(1.58), Q_m_ = 1.45 ± 0.065, narrower than the Inner Mongolia Autonomous Region specimens. Nevertheless, based on the phylogenetic analyses and morphological characteristics, we consider the specimens from Shandong Province and Inner Mongolia Autonomous Region to represent the same species. Given the comparatively more humid climate of Shandong Province and the observation that the Shandong specimen appeared to be at an earlier developmental stage, we hypothesise that climatic conditions and developmental stages may influence basidiospore dimensions. Further, more specimens from diverse regions are required to substantiate this hypothesis.

### Crepidotus stenocystis

Pouzar, 2005

92BF45A8-7F81-596F-B2B3-C5B77FBD0BA6

#### Materials

- **Type status:** Other material. **Occurrence:** occurrenceID: 5B34AB0C-F649-5C02-B6B6-BFA9E9FD2C98; **Taxon:** kingdom: Fungi; phylum: Basidiomycota; class: Agaricomycetes; order: Agaricales; family: Crepidotaceae; genus: Crepidotus; taxonRank: species; **Location:** country: China; stateProvince: Inner Mongolia Autonomous Region; county: Chifeng City; verbatimLocality: Hexigten Banner, Bayan Aobao National Nature Reserve; **Identification:** identifiedBy: TieZhi Liu; **Event:** year: 2007; month: August; day: 21; **Record Level:** institutionID: CFSZ; collectionID: CFSZ 3210

#### Description

**Figure 4.**
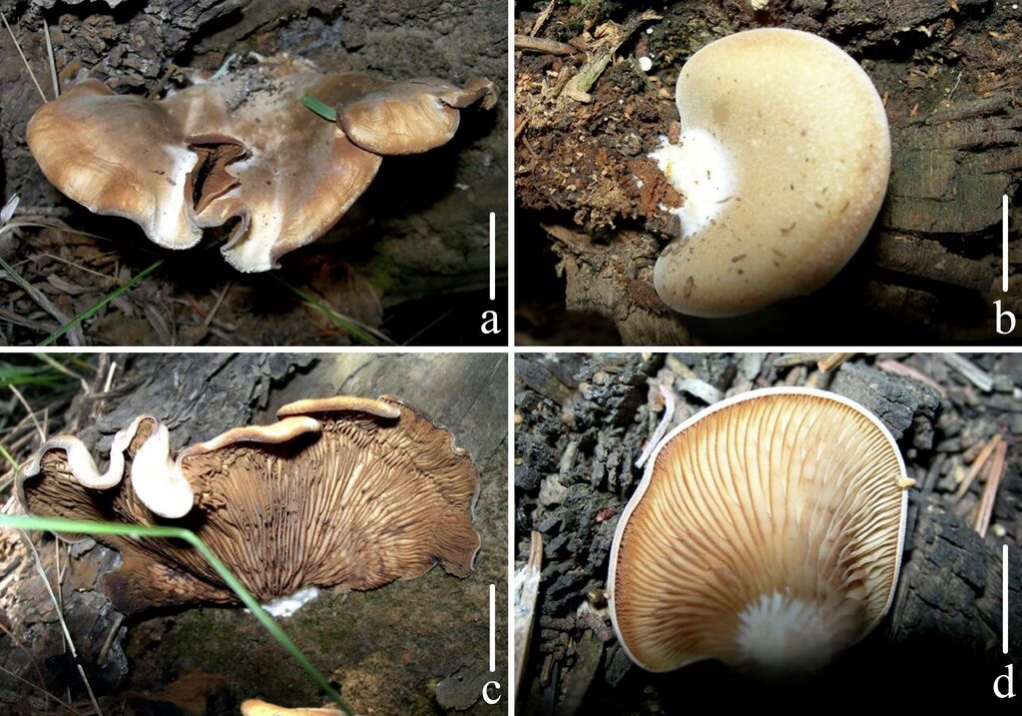
Fresh basidiomata of *Crepidotusstenocystis*. Bars: **a**–**d** = 5 mm. Photos by Tiezhi Liu.

**Pileus** 10–30 (90) mm, attached laterally, semicircular when young, White (LIII) to *Cream Colour (XVI19'*f*), when mature flabelliform to petaloid, nearly applanate, Old Gold (XVI19'*i*) to Buffy Citrine (XVI19'*k*), at times Saccardo's Olive (XVI19'*m*) by basidiospores depositing on surface, margin incurved in all stages; surface pubescent when young, silky when mature, dry, not hygrophanous, non-striated; dense pubescence near the point of attachment. **Lamellae** 15–25 mm, *L* = 19–27, *l* = 3–7, adnexed to subdecurrent, subventricose, edge smooth, *Cream Colour (XVI19'*f*), edge *Wood Brown (XL17''') to Buffy Brown (XL17'''*i*). **Stipe** as a lateral knob, White (LIII). **Context** White (LIII), thicker near the base, fragile in texture. **Odour** and **taste** not distinctive. (Fig. 4)

**Figure 5.**
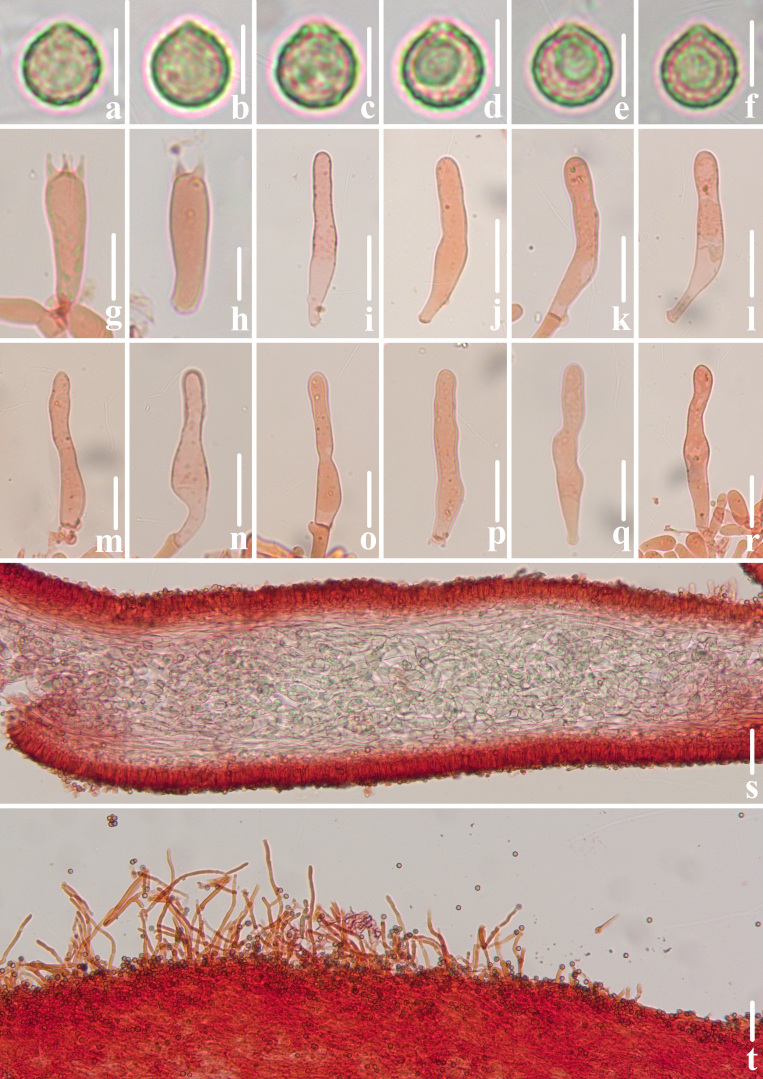
Microscopic features of *Crepidotusstenocystis* (CFSZ 3210). **a**–**f** Lateral view of basidiospores; **g**, **h** Basidia; **i**–**r** Cheilocystidia; **s** Lamellae trama; **t** Pileipellis. Bars: **a**–**f** = 5 μm; **g**–**h** = 10 μm; **i**–**r** = 20 μm; **s**–**t** = 50 μm. Structures **a**–**f** were rehydrated in 5% KOH aqueous solution and **g**-**t** were stained in 1% Congo red aqueous solution. Anatomical examination by Menghui Han.

**Figure 6.**
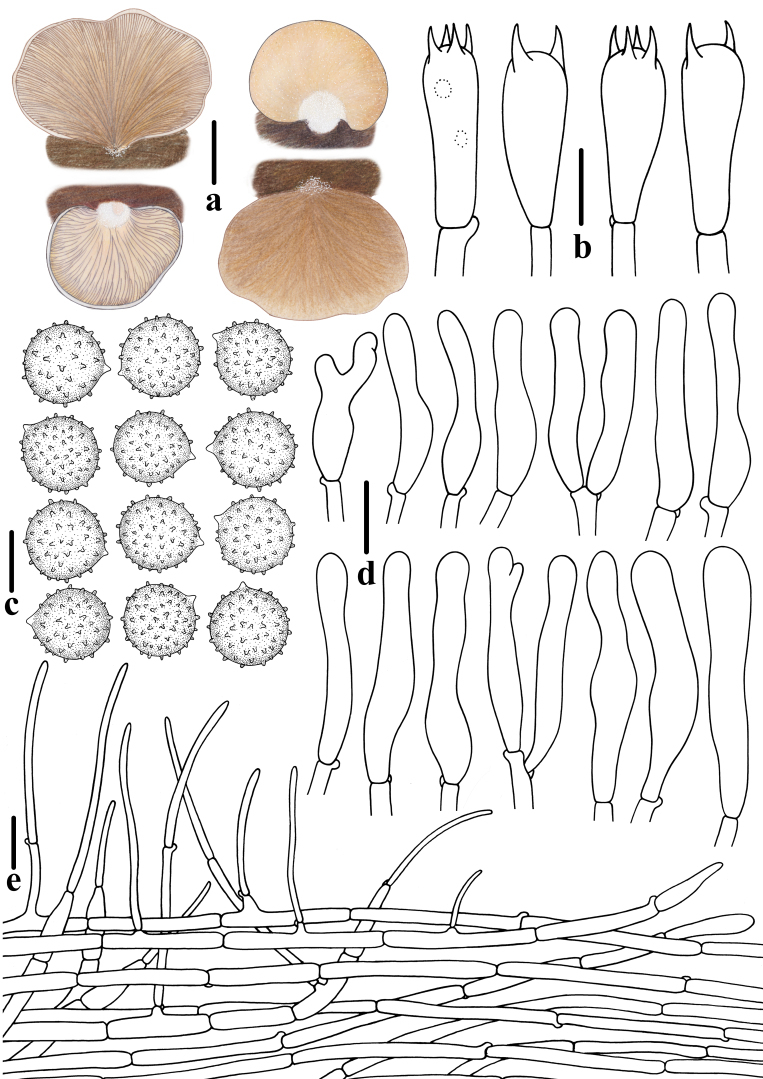
Morphological features of *Crepidotusstenocystis* (CFSZ 3210). **a** Basidiomata; **b** Basidiospores; **c** Basidia; **d**, **e** Cheilocystidia; **f** Pileipellis. Bars: **a** = 10 mm; **b**, **d** = 10 μm; **c** = 5 μm; **e** = 20 μm. Drawing by Menghui Han.

**Basidiospores** [110/3/1] (5.5)5.8–6.2–6.7(7.2) × (5.2)5.3–5.8–6.4(6.9) μm, Q = (1.01)1.02–1.13(1.15), Q_m_= 1.08 ± 0.03, globose to nearly globose in lateral view, yellowish-brown in 5% KOH aqueous solution, verrucose (ornamentation type IV, Giovanni (2008)), usually containing an oil drop (under oil). **Basidia** 20–27 × 6.8–9.5 μm, clavate, 4–spored, rarely 2–spored, sterigmata 2.4–5.7 μm long, thin-walled (< 0.5 μm thick), hyaline. **Pleurocystidia** absent. **Cheilocystidia** 40–66 × 7.3–10.2 μm, narrowly utriform to lageniform, elongated, sometimes near capitate, occasionally bifurcate, thin-walled (< 0.5 μm thick), hyaline. **Pileipellis** a trichoderm, composed of nearly parallel arranged cylindrical hyphae, 6–11 μm diam., thin-walled; the terminal or middle cells sometimes differentiate into slender cylindrical hyphae, 3–6 μm diam., colourless to yellowish, oblique to erect, forming pubescence in pileus surface. **Lamellae trama** irregular, composed of interwoven hyphae, 10–21 μm diam. Clamp connections present in all tissues (Figs 5, 6).

#### Distribution

China, Czechia, Slovakia, Finland, Switzerland, Germany, United States.

#### Ecology

Solitary or scattered on decaying wood or dead branches of *Piceameyeri*.

#### Notes

According to the classification of Consiglio and Setti, *C.stenocystis* is classified as C.subg.Dochmiopussect.Sphaeruli, based on its subglobose, verrucose basidiospores and the presence of clamp connections (Consiglio and Setti 2008). Within this section, *C.stenocystis* closely resembles *C.applanatus* (Pers.) P. Kumm., but *C.applanatus* differs by possessing translucent pileus, white lamellae edge and a cutis pileipellis (Senn-Irlet 1995). Initial studies of *C.stenocystis* were based on European specimens, with the species first described by Zdeněk Pouzar in 2005 from specimens collected in the Czech Republic (now Czechia) (Pouzar 2005). Subsequently, Consiglio and Setti considered *C.stenocystis* to be a synonym of C.malachiusvar.trichifer (*C.malachius* Sacc.) (Consiglio and Setti 2008). However, based on combined phylogenetic and morphological analyses, Jančovičová et al. treated *C.stenocystis* as a distinct and valid taxonomic different from *C.malachius*; we also agree with their view (Jančovičová et al. 2017). A notable macroscopic difference has been observed between European and Chinese specimens. According to the original descriptions of the European specimens, Jančovičová et al. considered the pileus of *C.stenocystis* to be hygrophanous (Jančovičová et al. 2017). In contrast, our examination of Chinese specimens indicate a non-hygrophanous pileus. Despite this discrepancy, we regard our specimens as *C.stenocystis*, attributing the observed difference to environmental factors. Previous studies have noted *C.stenocystis* prefers humid climates, whereas our specimens were collected from the Inner Mongolia Autonomous Region, a region characterised by a comparatively arid climate, which may cause the non-hygrophanous pileus (Pouzar 2005, Jančovičová et al. 2017). In the phylogenetic tree, our sequence clustered with European specimens in a well-supported clade. Additionally, Jančovičová et al. described *C.stenocystis* possessing pileocystidia to be terminal cells that have not yet differentiated into slender cylindrical hyphae. Our specimen is similar to those studied by Jančovičová et al. in other macroscopical and microscopical characteristics, especially basidiospore and cheilocystidia.

## Identification Keys

### Key to *Crepidotus* species of the Inner Mongolia Autonomous Region

**Table:** 

1	Pileus yellowish-brown	2
–	Pileus not yellowish-brown	3
2	Lamellae edge smooth, brown	*C.stenocystis*
–	Lamellae edge fimbriate, white	*C.crocophyllus*
3	Basidiospores globose to broadly ellipsoid, Q < 1.30	*C.cesatii*
–	Basidiospores ellipsoid to slightly amygdaliform, Q > 1.30	4
4	Clamp connections absent	5
–	Clamp connections present	6
5	Pileus margin serrate at maturity	*C.dentatus*
–	Pileus margin integral at maturity	*C.mollis*
6	Basidiospores smooth	*C.cinereofuscus*
–	Basidiospores ornamented	*C.subverrucisporus*

## Analysis

### Phylogenetic analysis

**Figure 7.**
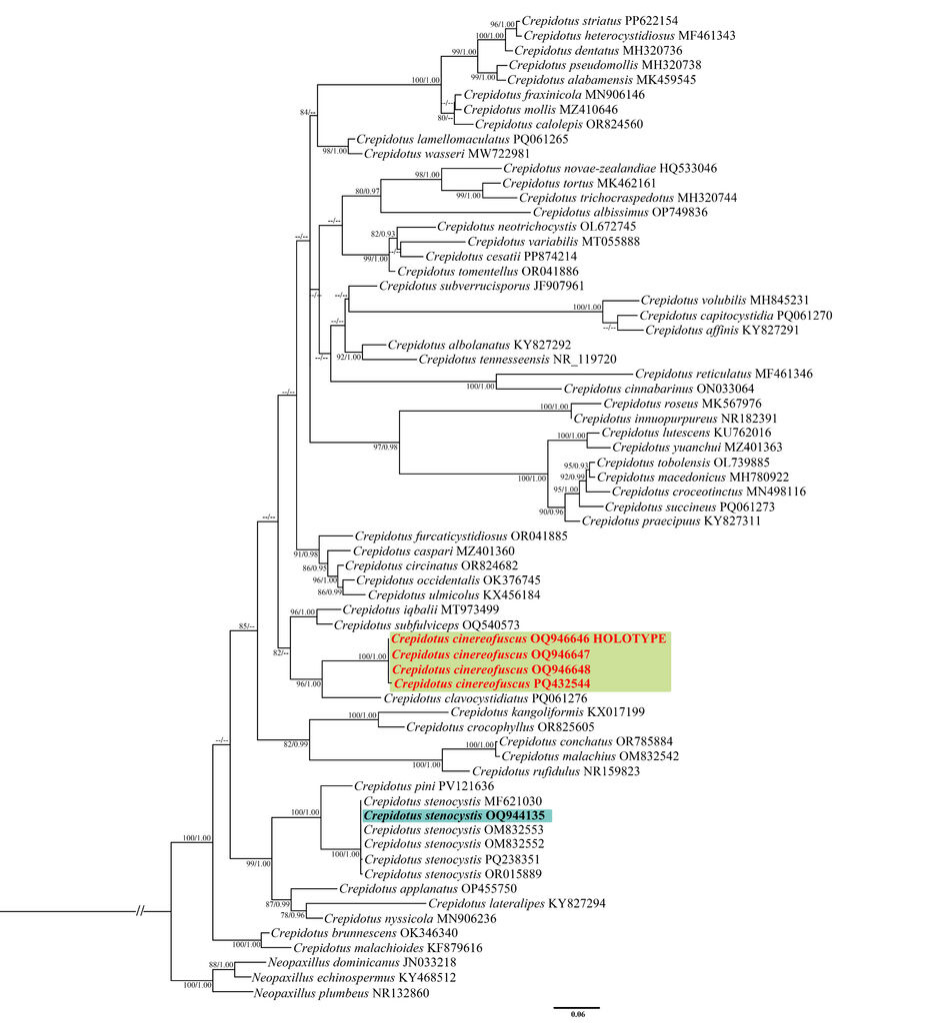
Bayesian tree inferred from ITS sequences showing phylogenetic relationships of a new species and a new recorded species for China. Bootstrap support values (BS) over 75% and Bayesian posterior probabilities (BPP) over 0.90 are indicated. The new species is highlighted in red and the new recorded species is indicated in bold.

A total of 119 ITS sequences were downloaded from GenBank and five sequences were amplified in this study. *Neopaxillusdominicanus* Angelini & Vizzini, *N.echinospermus* (Speg.) Singer and *N.plumbeus* Singer & Lodge were selected as the outgroup (Vizzini et al. 2012). The aligned ITS dataset matrix included 863 sites (including gaps). The best-fit substitution models for Bayesian Inference and Maximum Likelihood analyses were GTR + I + G and TVM + I + G4m, respectively. For the BI analyses, the average standard deviation of frequencies = 0.00764, the effective sample size (ESS) values for all parameters exceeded 200 and the Potential Scale Reduction Factor (PSRF) parameter values = 1.000 after 2,000,000 MCMC generations. For the ML analyses, the final log-likelihood value was -10876.72. The BI and ML analyses trees exhibited similar topologies and the BI tree is shown in Fig. 7.

The new species *C.cinereofuscus* is phylogenetically related to *C.clavocystidiatus*, but forms a distinct and well-supported clade clearly separated from *C.clavocystidiatus*. In addition, the two species exhibit significant morphological differences: *C.clavocystidiatus* is characterised by a white to light yellow pileus and faintly verrucose basidiospores, whereas *C.cinereofuscus* has a silver-grey to pale brownish-grey pileus and smooth basidiospores. Notably, *C.cinereofuscus* clusters with *C.clavocystidiatus*, rather than grouping with other species that also possess smooth basidiospores and clamp connections. This situation may be attributed to two possible factors: first, the limited number of gene regions employed may have resulted in insufficient sequence data; second, the molecular phylogenetic framework of *Crepidotus* is in conflict with the classifications proposed by Hesler & Smith and Consiglio & Setti, both of which were based on morphological characteristics (Aime 2001, Han et al. 2024). Future studies should incorporate additional gene regions and more detailed morphological examinations to establish a more natural classification.

*Crepidotusstenocystis* clusters with *C.applanatus*–group, which is characterised by globose basidiospores covered by echinulate or verrucose ornamentation. The result is consistent with both phylogenetic expectations and previous studies (Jančovičová et al. 2017, Jančovičová et al. 2024). Within *C.stenocystis*, the sequence “OQ944135” from a Chinese specimen clusters with European sequences in a strongly-supported clade, indicating phylogenetic congruence between Chinese and European material. According to the classification proposed by Consiglio and Setti (2008), *C.cinereofuscus* is classified as C.subg.Dochmiopussect.Autochthoni, while *C.stenocystis* belongs to C.subg.Dochmiopussect.Sphaeruli. Although *C.cinereofuscus* and *C.stenocystis* are classified within the same subgenus, they form separate and well-supported clades in the phylogenetic tree.

## Discussion

This study reports the first record of one *Crepidotus* species from China and describes one new species, both of which are well-supported in the phylogenetic tree. The new species, *C.cinereofuscus*, is particularly notable for its smooth basidiospores and the presence of clamp connections, supporting its classification in C.subg.Dochmiopussect.Autochthoni. This section represents a distinctive group within *Crepidotus*. Currently, this section contains only eight species, amongst which *C.cinereofuscus*, *C.trichocraspedotus* and *C.lamellomaculatus* have been published, based on Chinese specimens (Ge and Bau 2020, Han et al. 2024). As more and more taxa within C.subg.Dochmiopussect.Autochthoni are discovered and described, our understanding of the relationship between clamp connections and smooth basidiospores is expected to deepen.

Historically, species identification within *Crepidotus* has relied primarily on morphological characteristics, due to the limited availability of molecular sequence data (Consiglio and Setti 2008). This may led to misidentification. For example, *C.stenocystis* may have been misidentified within the *C.applanaus*–group, as both species share similar basidiospores and cheilocystidia, which are key diagnosis characteristics in species identification (Senn-Irlet 1995, Pouzar 2005, Consiglio and Setti 2008). The application of molecular techniques has significantly enhanced the accuracy of species delimitation, clarified taxonomic boundaries amongst closely-related taxa and facilitated the discovery of numerous new taxa.

## Supplementary Material

XML Treatment for Crepidotus cinereofuscus

XML Treatment for Crepidotus stenocystis

## Figures and Tables

**Table 1.** Information on the sequences used in this study. The new amplified sequences in this study are shown in bold.

**Species**	**Voucher/Strain no.**	**Location**	**ITS Sequence no.**	**Reference**
*C.affinis*	PDD:72848	New Zealand	KY827291	Horak (2018)
*C.alabamensis*	TBGT15610	India	MK459545	Kumar et al. (2022)
*C.albissimus*	S.D. Russell ONT WPMC iNaturalist 137604449	USA	OP749836	Direct Sub.
*C.albolanatus*	PDD:72865	New Zealand	KY827292	Horak (2018)
*C.applanatus*	S.D. Russell ONT iNaturalist # 98660416	USA	OP455750	Direct Sub.
*C.brunnescens*	MO414862	USA	OK346340	Direct Sub.
*C.calolepis*	iNaturalist 142786935	USA	OR824560	Unpublished
*C.capitocystidia*	FFAAS1310	China	PQ061270	Han et al. (2024)
*C.caspari*	FFAAS0343	China	MZ401360	Na et al. (2022)
*C.cesatii*	SLO 2607	Slovakia	PP874214	Jančovičová et al. (2024)
***C.cinereofuscus***	**CFSZ 24821**	**China**	**OQ946646**	**This study**
***C.cinereofuscus***	**CFSZ 24821**	**China**	**OQ946647**	**This study**
***C.cinereofuscus***	**CFSZ 24838**	**China**	**OQ946648**	**This study**
***C.cinereofuscus***	**FFAAS0367**	**China**	**PQ432544**	**This study**
*C.cinnabarinus*	S.D. Russell iNaturalist # 25244686	USA	ON033064	Direct Sub.
*C.circinatus*	iNaturalist 141511315	USA	OR824682	Direct Sub.
*C.clavocystidiatus*	FFAAS1316	China	PQ061276	Han et al. (2024)
*C.croceotinctus*	iNat31834012	USA	MN498116	Direct Sub.
*C.crocophyllus*	OMDL K. Canan iNaturalist # 170504506	USA	OR825605	Direct Sub.
*C.conchatus*	OMDL K. Canan iNaturalist # 170504453	USA	OR785884	Unpublished
*C.dentatus*	HMJAU37097	China	MH320736	Ge and Bau (2020)
*C.fraxinicola*	S.D. Russell MycoMap # 5922	USA	MN906146	Direct Sub.
*C.furcaticystidiosus*	FFAAS1027	China	OR041885	Liu et al. (2024)
*C.heterocystidiosus*	HMJAU37045	China	MF461343	Ge and Bau (2020)
*C.innuopurpureus*	MEL 2503290	Australia	NR182391	Unpublished
*C.iqbalii*	LAH36655	Pakistan	MT973499	Izhar et al. (2021)
*C.kangoliformis*	BAP 664	São Tomé	KX017199	Desjardin and Perry (2016)
*C.lamellomaculatus*	FFAAS1305	China	PQ061265	Han et al. (2024)
*C.lateralipes*	PDD:72571	New Zealand	KY827294	Horak (2018)
*C.lutescens*	HMJAU 21976	China	KU762016	Ge (2017)
*C.macedonicus*	DB3859	Hungary	MH780922	Unpublished
*C.malachioides*	WU 31421	Austria	KF879616	Direct Sub.
*C.malachius*	SLO 2394	Slovakia	OM832542	Jančovičová et al. (2022)
*C.mollis*	IBL17	Poland	MZ410646	Unpublished
*C.neotrichocystis*	CS1150	Malta	OL672745	Sammut (2021)
*C.novae-zealandiae*	PDD:95850	New Zealand	HQ533046	Direct Sub.
*C.nyssicola*	S.D. Russell MycoMap # 7399	USA	MN906236	Direct Sub.
*C.occidentalis*	MUOB:367585	USA	OK376745	Direct Sub.
*C.praecipuus*	PDD:72481	New Zealand	KY827311	Horak (2018)
*C.pseudomollis*	HMJAU37125	China	MH320738	Ge and Bau (2020)
*C.pini*	MJ23-006	France	PV121636	Unpublished
*C.reticulatus*	HMJAU37089	China	MF461346	Direct Sub.
*C.roseus*	TBGT15507	India	MK567976	Kumar et al. (2022)
*C.rufidulus*	PDD 98272	New Zealand	NR159823	Horak (2018)
*C.stenocystis*	PRM911279	Czech Republic	MF621030	Jančovičová et al. (2017)
*C.stenocystis*	SLO 481	Slovakia	OM832552	Jančovičová et al. (2022)
*C.stenocystis*	SLO 2557	Slovakia	OM832553	Jančovičová et al. (2022)
***C.stenocystis***	**CFSZ 3210**	**China**	**OQ944135**	**This study**
*C.stenocystis*	STU:SMNS-STU-F-0900915	Germany	OR015889	Direct Sub.
*C.stenocystis*	NSK 1017346	Russia	PQ238351	Direct Sub.
*C.striatus*	FCATAS5680	China	PP622154	Direct Sub.
*C.subfulviceps*	HFS iNaturalist # 91902833	USA	OQ540573	Direct Sub.
*C.subverrucisporus*	15720	Italy	JF907961	Direct Sub.
*C.succineus*	FFAAS1313	China	PQ061273	Han et al. (2024)
*C.tennesseensis*	LRH29144	USA	NR_119720	Horak et al. (2015)
*C.tobolensis*	LE313671	Russia	OL739885	Malysheva et al. (2022)
*C.tomentellus*	FFAAS1025	China	OR041886	Liu et al. (2024)
*C.tortus*	TBGT17194	India	MK462161	Kumar et al. (2022)
*C.trichocraspedotus*	HMJAU37250	China	MH320744	Ge and Bau (2020)
*C.ulmicolus*	HMJAU37027	China	KX456184	Zhang et al. (2022)
*C.variabilis*	SLO 2016	Slovakia	MT055888	Jančovičová et al. (2020)
*C.wasseri*	LE 287679	Russia	MW722981	Kapitonov VI (2021)
*C.volubilis*	TBGT15648	India	MH845231	Kumar et al. (2018)
*C.yuanchui*	FFAAS0341	China	MZ401363	Na et al. (2022)
*N.dominicanus*	F 1059091	Mexico	JN033218	Vizzini et al. (2012)
*N.echinospermus*	AH45884	Brazil	KY468512	Unpublished
*N.plumbeus*	F 1068564	Puerto Rico	NR132860	Vizzini et al. (2012)

## References

[B12532402] Aime M. Catherine (2001). Biosystematic studies in *Crepidotus* and the Crepidotaceae (Basidiomycetes, Agaricales). Virginia Polytechnic Institute and State University, Blacksburg,.

[B12532669] Alzohairy Ahmed Mansour (2011). BioEdit: An important software for molecular biology. GERF Bulletin of Biosciences.

[B12963948] Bai H., Jiang H., Cong L., Shi D. M., Lin R. J., Di J. L. (2022). Diversity of macrofungi in different broad-leaved forests in Gaogestai Hanwula Nature Reserve. Journal of Zhejiang A&F University.

[B12532355] Consiglio Giovanni, Setti Ledo (2008). Il genere *Crepidotus* in Europa.

[B12532705] Desjardin DE., Perry BA. (2016). Dark-spored species of Agaricineae from Republic of São Tomé and Príncipe, West Africa. Mycosphere.

[B12532590] Edler Daniel, Klein Johannes, Antonelli Alexandre, Silvestro Daniele (2021). raxmlGUI 2.0: A graphical interface and toolkit for phylogenetic analyses using RAxML. Methods in Ecology and Evolution.

[B12532491] Ge Yupeng (2017). Taxonomy and phylogeny of Crepidotaceae in China.

[B12532527] Ge Yupeng, Yang Sisi, Bau Tolgor (2017). *Crepidotuslutescens* sp. nov. (Inocybaceae, Agaricales), an ochraceous salmon colored species from northeast of China. Phytotaxa.

[B12532385] Ge Yupeng, Bau Tolgor (2020). Descriptions of six new species of *Crepidotus* from China. Mycosystema.

[B12532722] Hall T. A., (1999). BioEdit: A user-friendly biological sequence alignment editor and analysis program for Windows 95/98/NT. Nucleic Acids Syiesmposium Ser.

[B12532757] Han Menghui, Na Qin, Wei Renxiu, Zeng Hui, Hu Yaping, Zhang Libo, Du Jinhong, Zou Li, Tang Weimin, Cheng Xianhao, Ge Yupeng (2024). Phylogenetic and morphological perspectives on Crepidotussubg.Dochmiopus: Exploratively unveiling hidden diversity in China. Journal of Fungi.

[B12532394] Hesler L. R., Smith A. H. (1965). North American species of *Crepidotus*.

[B12532687] Horak Egon, Matheny P. Brandon, Desjardin Dennis E., Soytong Kasim (2015). The genus *Inocybe* (Inocybaceae, Agaricales, Basidiomycota) in Thailand and Malaysia. Phytotaxa.

[B12532731] Horak Egon (2018). Fungi of New Zealand volume 6 Agaricales (Basidiomycota) of New Zealand 2 brown spored genera.

[B12532444] Izhar A, Usman M, Khalid B N (2021). *Crepidotusiqbalii* (Crepidotaceae, Agaricales) a new stipitate species, from Pakistan. Phytotaxa.

[B12532372] Jančovičová Soňa, Adamčík Slavomír, Looney Brian P., Caboň Miroslav, Čaplovičová Mária, Kopáni Martin, Pennycook Shaun R., Adamčíková Katarína (2017). Delimitation of European *Crepidotusstenocystis* as different from the North American species *C.brunnescens* (Inocybaceae, Agaricales). Phytotaxa.

[B12532508] Jančovičová Soňa, Adamčíková Katarína, Caboň Miroslav, Adamčík Slavomír (2020). How variable is *Crepidotusvariabilis*?. Phytotaxa.

[B12532411] Jančovičová Soňa, Adamčíková Katarína, Caboň Miroslav, Adamčík Slavomír (2022). Phylogeny of *Crepidotusapplanatus* look-alikes reveals a convergent morphology evolution and a new species *C.pini*. Journal of Fungi.

[B12532739] Jančovičová Soňa, Adamčíková Katarína, Caboň Miroslav, Graddy Mary G., Matheny P. Brandon, Noffsinger Chance R., Wheeler Tim B., Adamčík Slavomír (2024). Taxonomic reintroduction of the holarctic saprotrophic fungus *Crepidotuscinnamomeus*. Mycological Progress.

[B12532572] Kapitonov VI (2021). *Crepidotuswasseri* Kapitonov, Biketova, Zmitrovich & Á. Kovács, sp. nov.. Persoonia - Molecular Phylogeny and Evolution of Fungi.

[B12532660] Katoh K., Misawa K., Kuma K., Miyata T. (2002). MAFFT: A novel method for rapid multiple sequence alignment based on fast Fourier transform. Nucleic Acids Research.

[B12532773] Katoh Kazutaka, Standley Daron M. (2016). A simple method to control over-alignment in the MAFFT multiple sequence alignment program. Bioinformatics.

[B13250971] Kozlov AM, Darriba D, Flouri T (2019). RAxML-NG: a fast, scalable and user-friendly tool for maximum likelihood phylogenetic inference. Bioinformatics.

[B12532545] Krisai-Greilhuber Irmgard, Senn-Irlet Béatrice, Voglmayr Hermann (2002). Notes on *Crepidotus* from Mexico and the South-eastern USA. Persoonia - Molecular Phylogeny and Evolution of Fungi.

[B12532536] Kumar A Manoj, Vrinda KB., Pradeep CK. (2018). Two new species of *Crepidotus* (Basidiomycota, Agaricales) from peninsular India.. Phytotaxa.

[B12534140] Kumar A. Manoj, Pradeep C. K., Aime M. Catherine (2022). New species and new records of *Crepidotus* (Crepidotaceae) from India. Mycological Progress.

[B12532581] Liu Peigui (1995). Five new species of Agricales from southern and southeastern Yunnan, China. Mycotaxon.

[B12532599] Liu Shi-Liang, Zhao Peng, Cai Lei, Shen Shan, Wei Hao-Wen, Na Qin, Han Menghui, Wei Renxiu, Ge Yupeng, Ma Haixia, Karunarathna Samantha Chandranath, Tibprommab Saowaluck, Zhang Bo, Dai Dan, Lin Lu, Fan Xin-Lei, Luo Zong-Long, Shen Hong-Wei, Lu Li, Lu Wenhua, Xu Rui-Fang, Tohtirjap Ablat, Wu Fang, Zhou Li-Wei (2024). Catalogue of fungi in China 1. New taxa of plant-inhabiting fungi. Mycology.

[B12532696] Malysheva Ekaterina F., Kiyashko Anna A., Malysheva Vera F., Shikalova Elena A. (2022). A survey of rare species of agaricoid fungi (Basidiomycota) from South Siberia, Russia. Turczaninowia.

[B12532453] Matheny P. Brandon, Curtis Judd M., Hofstetter Valérie, Aime M. Catherine, Moncalvo Jean-Marc, Ge Zai-Wei, Yang Zhu-Liang, Slot Jason C., Ammirati Joseph F., Baroni Timothy J., Bougher Neale L., Hughes Karen W., Lodge D. Jean, Kerrigan Richard W., Seidl Michelle T., Aanen Duur K., DeNitis Matthew, Daniele Graciela M., Desjardin Dennis E., Kropp Bradley R., Norvell Lorelei L., Parker Andrew, Vellinga Else C., Vilgalys Rytas, Hibbett David S. (2006). Major clades of Agaricales: a multilocus phylogenetic overview. Mycologia.

[B12532517] Na Qin, Liu Zewei, Zeng Hui, Cheng Xianhao, Ge Yupeng (2022). *Crepidotusyuanchui* sp. nov. and *C.caspari* found in subalpine areas of China. Mycoscience.

[B12532628] Na Qin, Zeng Hui, Hu Yaping, Ding Hui, Ke Binrong, Zeng Zhiheng, Liu Changjing, Cheng Xianhao, Ge Yupeng (2024). Morphological and phylogenetic analyses reveal five new species of Porotheleaceae (Agaricales, Basidiomycota) from China. MycoKeys.

[B12532678] Nylander Johan A. A., Wilgenbusch James C., Warren Dan L., Swofford David L. (2008). AWTY (are we there yet?): a system for graphical exploration of MCMC convergence in Bayesian phylogenetics. Bioinformatics.

[B12532554] Pouzar Zdeněk (2005). Notes on some European species of the genus *Crepidotus* (Agaricales).. Czech Mycology.

[B12964176] Rambaut A., Drummond A. J., Xie D., Baele G., M.A Suchard (2018). Posterior Summarization in Bayesian Phylogenetics Using Tracer 1.7. Systematic Biology.

[B12532714] Ridgway R, (1912). Color standards and color nomenclature.

[B12532651] Ronquist Fredrik, Huelsenbeck John P. (2003). MrBayes 3: Bayesian phylogenetic inference under mixed models. Bioinformatics.

[B12532782] Sammut Carmel (2021). Further additions to the mycobiota of malta. Ecologia mediterranea.

[B12532499] Senn-Irlet (1995). The genus *Crepidotus* (Fr.) Staude in Europe. Persoonia.

[B12963938] Shi D. M. (2021). A Preliminary Study on Macrofungal Resources in Shariwendu Nature Reserve, Inner Mongolia.

[B12534149] Tao X. (2021). Macrofungal diversity in Houhe National Nature Reserve.

[B12532363] Vizzini Alfredo, Angelini Claudio, Ercole Enrico (2012). A new *Neopaxillus* species (Agaricomycetes) from the Dominican Republic and the status of *Neopaxillus* within the Agaricales. Mycologia.

[B12532346] Wei Tiezheng, Yao Yijian (2009). Literature review of *Crepidotus* species in China. Journal of Fungal Research.

[B12964152] White T. J., Bruns T. D., Lee S. B., Taylor J. W. (1990). Amplification and direct sequencing of Fungal Ribosomal RNA Genes for phylogenetics.

[B12532483] P Zhang (2017). Diversity of macrofungi in the Greater and Lesser Khinggan Moutains.

[B12532642] Zhang Peng, Ge Yupeng, Bau Tolgor (2022). Two new species of *Crepidotus* (Crepidotaceae) from China. Phytotaxa.

